# Social burden of three major diseases in Japan: A time trend and future projections using the comprehensive cost of illness method

**DOI:** 10.1371/journal.pone.0280311

**Published:** 2023-01-11

**Authors:** Koki Hirata, Kunichika Matsumoto, Yosuke Hatakeyama, Ryo Onishi, Kanako Seto, Tomonori Hasegawa

**Affiliations:** Department of Social Medicine, Toho University School of Medicine, Tokyo, Japan; Indian Institute of Technology Jodhpur, INDIA

## Abstract

**Background:**

Three major diseases in Japan, cancer, heart disease, and cerebrovascular disease (CVD) are the leading causes of death in Japan. This study aimed to clarify the social burden of these diseases, including long-term care (LTC), and to predict future trends.

**Methods:**

The comprehensive cost of illness (C-COI), a modification of the cost of illness (COI), was used to estimate the social burden of the three major diseases in Japan. The C-COI can macroscopically estimate both direct and indirect costs, including the LTC. A new method for future projections of the C-COI was developed according to the method for future projections of the COI. All data sources were government statistics.

**Results:**

The C-COI of cancer, heart diseases, and CVD in 2017 amounted to 11.0 trillion JPY, 5.3 trillion JPY, and 6.5 trillion JPY, respectively. The projected future C-COI in 2029 was 10.3 trillion JPY, 5.3 trillion JPY, and 4.4 trillion JPY, respectively. In 2029, the LTC costs accounted for 4.4%, 12.8%, and 44.1% of the total C-COI, respectively. Informal care costs are projected to be approximately 1.7 times higher, assuming that all family caregivers will be replaced by professional caregivers in 2029.

**Conclusion:**

Indirect costs for all three diseases were projected to decrease owing to aging of the patient. In contrast to the other two diseases, the LTC cost of CVD accounted for a large proportion of the burden. The burden of CVD is expected to decrease in the future, but informal care by older family caregivers is suggested to reach its limits. In the future, the focus of resource allocation should shift from medical care to LTC, especially support for family caregivers. A method of future projections for the social burden based on the C-COI was considered effective for identifying issues for healthcare policy in the context of the times.

## Introduction

The three major diseases in Japan that have been the leading causes of death since the 1950s are cancer (ICD-10 codes C00–C97, D00–D09), heart disease (I01–I02.0, I05–I09, I20–I25, I27, I30–I52), and cerebrovascular disease (CVD) (I60–I69). Each of these diseases is strongly associated with aging. Since 1981, the consistently increasing incidence of cancer has been a preponderant cause of death in Japan. The second leading cause of death is heart disease, which is also increasing. In 2019, CVD was ranked fourth among the causes of death. It was the most common cause of death until the 1970s but has been declining since then. The deaths caused by these diseases accounted for 27.3%, 15.0%, and 8.8% of all deaths, respectively; the national medical expenses accounted for 4.0 trillion yen (36.0 billion dollars, 110 JPY = 1 US$), 2.0 trillion yen (18.6 billion dollars), and 1.8 trillion yen (13.4 billion dollars) in 2019, respectively [1]. It was assumed that each of these three diseases had different characteristics and that the structure of their social burden also differed.

Japan is the world’s most aged nation. In 2017, 27.7% of Japanese people were aged 65 years or older, and 13.8% were aged 75 years or older [2]. Aging is changing the disease structure in Japan, making chronic diseases important and acute diseases relatively insignificant [3]. Older adults are likely to have multiple chronic diseases and often require long-term care (LTC). In fact, the number of persons certified by the public LTC insurance system to receive assistance with the costs of using LTC services has doubled in the past 18 years, from 3.3 million in 2003 to 6.6 million in 2021 in Japan [4]. Therefore, the burden of diseases that require LTC can be underestimated if only the burden of acute care, such as treatment and medication, is considered [5]. In an aging society, it is desirable to comprehensively estimate the social burden of disease, including indirect and LTC costs, when estimating healthcare demand as a reference for formulating health policy.

Disability Adjusted Life Years (DALYs) are well-known estimates of the social burden of disease. DALYs are continuously being estimated on a global scale by the Global Burden of Disease (GBD) study led by the Institute for Health Metrics and Evaluation (IHME) [6]. The GBD study provided DALYs of the three major diseases from 1990 to the present, which showed that DALYs of cancer were on the rise but have levelled off since 2010, while DALYs of heart diseases and CVD have remained largely unchanged since 1990 [7]. Future projections of DALYs have also been attempted several times in GBD studies [8–10]. DALYs are a method of estimating the total quality of life and life expectancy lost by individuals with a disease, and may be useful for understanding the health loss of a country’s population. At the same time, other methods are needed to estimate the overall burden borne by society as a whole, including not only the patients themselves but also those around them. For example, DALYs cannot estimate the costs paid by society through the provision of LTC. In addition, because DALYs are indicators expressed in terms of ’years’, it can be difficult for non-specialist policy-makers and the general public to understand their meaning or to intuitively compare them with the costs of social problems in other sectors.

To overcome these problems, this study estimates the social burden of diseases using the comprehensive cost of illness (C-COI) method, which is an extension of the cost of illness (COI) method [5, 11–13]. Matsumoto et al. developed the C-COI method to include LTC costs, whereas the original COI method only estimated medical costs [5]. The COI was proposed by Rice to estimate the social burden of diseases and has been extensively used worldwide because it can be easily evaluated based on available government statistical data [14–23]. One of the strengths of the COI method is that it provides a comprehensive estimate of the social burden of diseases borne not only by the individual but also by society as a whole; the COI method can express the social burden of diseases in monetary terms at the macro level, taking into account both direct and indirect costs [11]. As costs are expressed in monetary terms, they are easy to understand and can be intuitively compared with costs in different sectors. In addition, since the COI method weighs indirect costs by sex and age, it can adjust for changes in demographic composition due to aging, making it suitable for estimating the social burden in an aging society.

In contrast, some studies have criticized the COI method, indicating that it is not clear whose “cost” it is. They further stated that it focuses on cost only and cannot compare the efficacy and utility of the treatment of diseases [24]. However, the COI method, which can comprehensively clarify the social burden of disease, may be useful in identifying priority interventions for health policies [25]. Moreover, the COI method can reveal which diseases and components of the social burden increase at that time. The C-COI method, which has the advantages of the original COI method and can also include LTC, can provide an important reference for healthcare policy in Japan, where the population is aging and the disease structure is changing from acute to chronic.

Our previous studies evaluated the historical changes in C-COI for the three major diseases and found that their total costs, composition, and trends over time varied widely [5, 13]. These studies also found that the proportion of LTC costs for CVD is larger than for the other two diseases, accounting for approximately half of the total costs. However, no previous studies have made future projections using the C-COI for the social burden of disease, including LTC. Future projections of the burden of major diseases, including indirect and LTC costs, using the C-COI method will contribute to the formulation of health policy in an aging society. This study aimed to project the future C-COI of three major diseases in Japan using newly developed methods.

## Materials and methods

### Data sources

All data used for past estimations and future projections of the C-COI were derived from government statistics that are publicly available [26]. The major government statistics used were the Patient Survey, Vital Statistics, Comprehensive Survey of Living Conditions, Statistics of Medical Care Activities in Public Health Insurance, and Statistics of Long-term Care Benefit Expenditures. Six other government statistics on the economy were also used. "Population Projections for Japan:2016 to 2065" by the National Institute of Population and Social Security Research were used as the population data [27]. Since some government statistics are surveyed every three years, the C-COI was also estimated every three years. However, other statistics have different survey years. Therefore, they were replaced according to the years of key statistics.

### Method of calculation for C-COI

The C-COI method was used to estimate past (in 2008, 2011, 2014, and 2017) and future values (in 2020, 2023, 2026, and 2029) of the social burden caused by the three major diseases in Japan.

The C-COI was defined as follows:

C-COI = MDC+FCC+MbC+MtC+ICC

[MDC: Medical direct cost, FCC: Formal care cost, MbC: Morbidity cost, MtC: Mortality cost, ICC: Informal care cost]

MDC, MbC, and MtC were the “medical costs” included in the original COI; FCC and ICC were the additional "LTC costs." MDC and FCC were direct costs, and MbC, MtC, and ICC were indirect costs.

In Japan, almost all medical costs, including those incurred in private hospitals, are covered by universal health coverage (UHC) and the associated patients’ out-of-pocket expenses. Therefore, this study defines MDC as the sum of outpatient and inpatient costs, which are both covered by UHC and out-of-pocket expenses. They can be calculated directly using government statistics related to UHC.

Since Japan introduced a public LTC insurance system in 2000, statistical data related to the system have been available for estimating LTC costs [28]. All older adults aged 65 years or older and those aged 45 to 64 years, who have diseases caused by aging, can use this public LTC insurance in case they need LTC services. Those in need of LTC services were requested to apply for certification. LTC approval boards then judge their nursing care level through screening (care levels 1–5 based on the assessment of care requirements) [29]. Nursing care level determines the upper limit of insurance benefits, and 90% of LTC service costs are reimbursed. FCC was defined as the cost of LTC services provided by public LTC insurance and associated out-of-pocket expenses.

FCC = NLR × LCy

[NLR: Number of public LTC insurance recipients, LCy: LTC costs per person per year]

MbC is the labor value lost during hospitalization or outpatient visits. It was calculated by multiplying the number of days of outpatient visits or hospitalization by the labor value by the sex–age group. Labor value includes both the value of domestic work and salary income. The calculation assumed that the patient had missed one day of work due to hospitalization and half a day for outpatient visits.

MbC = THD × LVd + TOVy/2 × LVd

[THD: total person-days of hospitalization, LVd: labor value per person per day, TOVy: total person-days dedicated to outpatient visits per year]

THD = HP × ALOS

[HP: Annual number of hospitalized patients, ALOS: Average length of hospital stay]

MtC was evaluated as the loss of human capital (human capital method) by multiplying the number of deaths from a disease by the lifetime labor value by the gender-age group. Lifetime labor value refers to the income that a patient who died from a disease would have earned in case they had survived to the average life span; it is calculated as the present value by accumulating the average income of the gender-age group. The discount rate for calculating the present value was 2% as recommended by Japan’s economic valuation guidelines [30].

MtC = NDy × LVl

[NDy: number of deaths per year; LVI: lifetime labor value per person]

ICC is a production value lost when people spend their time as a "family caregiver." The opportunity cost approach (OCA) was used for past ICC estimations. ICC by OCA was defined as the loss of labor value of family caregivers considering sex and age, which is similar to MbC.

ICC (OCA) = NFC × ATCd × 365 × LVh

[NFC: Number of family caregivers, ATCd: Average time for care per day, LVh:1-hour labor value per person]

### Methods of future projections

For future projections of medical costs, time-series forecasting with regression models, as established in previous studies, was used [17–20]. The necessary variables for the calculation of each component of medical costs were predicted from past government statistics using linear or logarithmic/exponential approximations. A previous study showed that using linear, logarithmic, and exponential approximations separately is more appropriate for combining variables with different trends than using a single approximation method [20]. It was expected that the future trend of each variable would eventually reach its limit and hit a “peak.” The principle is to use a logarithmic approximation for variables with an upward trend, an exponential approximation for variables with a downward trend, and a linear approximation for those with neither. However, this general rule was not adopted for the variables whose R-squared value was less than 0.3 in the logarithmic/exponential approximation. Instead, the average of the past values was used. All variables were projected by sex and age, multiplied by the estimated future population, and then added together. This approach has been adjusted for changes in demographic composition due to aging of the population.

The future MDC was calculated as follows:

MDC (future) = TOVy × OUC + THD × IUC

[OUC: outpatient unit cost; IUC: inpatient unit cost]

As mentioned earlier, the principle is to use a logarithmic approximation for variables with an upward trend and an exponential approximation for variables with a downward trend. However, unlike other variables, OUC and IUC were considered to have few factors that would reach their limits. In particular, the IUC for all three diseases has shown an exponential increase in recent years. Consequently, an exponential approximation was chosen. For the OUC, a linear approximation was selected considering past trends.

In this study, a new method was developed to project future LTC costs using time-series forecasting with a regression model. NLR, LCy, NFC, and ATCd were predicted using linear or logarithmic/exponential approximations, according to rules similar to those of medical costs. These variables were predicted by not only gender and age group but also nursing care level, as a ratio to the population. The labor value was highly dependent on economic conditions and was not predicted using this estimate. Therefore, the LVd, LVl, and LVh were not predicted. Values obtained in 2017 were used in this study.

Future projections of the ICC were made using not only the OCA, the standard method, but also the replacement approach (RA). In the RA, the average wage of professional caregivers was used instead of the lost family labor value through care.

ICC (RA) = NFC × ATCd × 365 × AWPC

[AWPC: Average wage of professional caregivers]

The study protocol was reviewed by the Ethical Committee of the Toho University School of Medicine. The committee confirmed that no approval was necessary for this kind of study in Japan owing to the anonymous nature of the data (reference number A19034).

## Results

**Fig 1:**
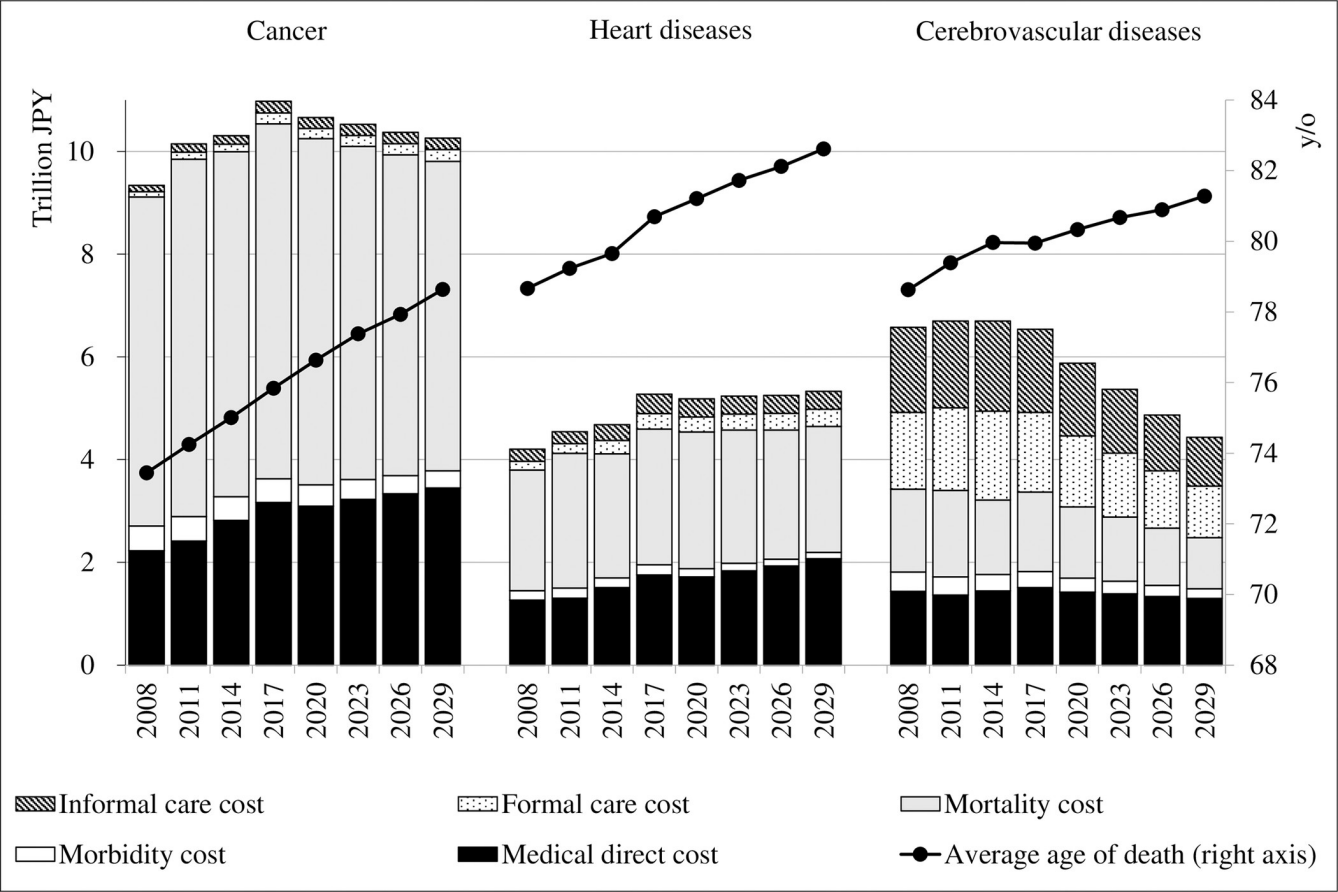
An overview of the results: Changes in the comprehensive cost of illness, past estimations and future projections.

Table 1 shows the changes in indicators related to the social burden of the three diseases, and Table 2 shows past and future estimates of the total amount of C-COI and the amount of each component for each disease. Future ICC projections are described for both the OCA and the RA. Fig 1 shows a visualization of the changes in the C-COI for each disease (Table 2). The ICC is represented by the OCA in Fig 1. The total amount of C-COI in 2017 was cancer, CVD, and heart disease, in that order. Regarding the composition ratios of the C-COI in 2017, cancer and heart disease had high proportions of medical costs (MDC, MbC, and MtC), with LTC cost proportions (FCC and ICC) of 4.0% and 12.9%, respectively. MtC accounted for the largest proportion of the medical costs of these two diseases (65.6% and 75.5%, respectively). The LTC costs of CVD were similar to those of medical costs, accounting for 48.5% of the total C-COI in 2017. The estimates showed that the FCC and ICC of CVD were similar. According to the OCA, ICC accounted for 51.1% of the LTC costs in 2017.

**Table 1:** Indicators related to the three major diseases, past values and future projections.

			2008	2011	2014	2017	2020	2023	2026	2029
Japan’s population*		(thousand)	127,692	127,799	127,083	126,706	125,325	123,751	121,903	119,850
	Proportion of older adults (65 and over)		22.1%	23.3%	26.0%	27.7%	28.9%	29.6%	30.2%	30.9%
			**Cancer**							
Estimated number of patients receiving medical treatment†		(thousand)	1,518	1,526	1,626	1,782	1,867	1,909	1,942	1,965
	Proportion of older adults		64.5%	65.9%	69.7%	74.5%	76.4%	77.4%	78.2%	78.9%
Average length of hospital stay†		(days)	23.9	20.6	19.9	17.1	14.6	12.9	11.3	10.0
Number of deaths†			342,963	357,305	368,103	373,334	389,180	397,623	400,831	408,497
	Proportion of older adults		79.1%	80.5%	83.7%	86.4%	87.4%	88.1%	88.7%	89.2%
Average age of death†		(y/o)	73.4	74.2	75.0	75.8	76.6	77.4	77.9	78.6
Inpatient unit cost (per person per day)†		(JPY)	27,869	31,631	37,017	43,515	48,524	55,322	63,071	71,906
Outpatient unit cost (per visit) †		(JPY)	15,192	16,535	18,605	21,176	21,672	23,019	24,366	25,713
The number of public LTC insurance recipients ‡			64,822	90,081	106,968	126,045	128,909	135,862	141,363	145,695
Average nursing care level†			2.83	2.45	2.33	2.52	2.36	2.35	2.34	2.33
			**Heart diseases**							
Estimated number of patients receiving medical treatment†		(thousand)	1,542	1,612	1,729	1,732	1,840	1,771	1,683	1,613
	Proportion of older adults		79.7%	80.4%	84.0%	86.5%	87.2%	87.8%	88.3%	88.8%
Average length of stay†		(days)	24.2	21.9	20.3	19.3	16.8	15.4	14.0	13.0
Number of deaths†			181,928	194,926	196,925	204,837	214,884	221,166	223,314	229,968
	Proportion of older adults		88.5%	89.6%	91.0%	92.7%	93.0%	93.4%	93.7%	94.1%
Average length of hospital stay†		(y/o)	78.7	79.2	79.7	80.7	81.2	81.7	82.1	82.6
Inpatient unit cost (per person per day) †		(JPY)	41,482	43,557	50,084	56,612	67,085	77,724	90,050	104,330
Outpatient unit cost (per visit) †		(JPY)	8,543	8,979	11,102	11,669	11,507	11,992	12,476	12,960
The number of public LTC insurance recipients ‡			152,154	160,514	208,569	244,098	230,661	236,568	240,176	242,080
Average nursing care level†			2.46	1.91	2.22	2.18	2.10	2.09	2.08	2.08
			**Cerebrovascular diseases**							
Estimated number of patients receiving medical treatment†		(thousand)	1,339	1,235	1,179	1,115	1,092	975	857	755
	Proportion of older adults		84.4%	85.2%	87.8%	86.5%	88.0%	87.8%	87.4%	87.1%
Average length of stay†		(days)	104.7	93	89.5	78.2	78.4	74.4	70.4	67.0
Number of deaths†			127,023	123,867	114,207	109,880	102,034	94,620	85,889	79,208
	Proportion of older adults		88.8%	89.6%	90.8%	91.4%	91.7%	91.8%	91.7%	91.9%
Average length of hospital stay†		(y/o)	78.6	79.4	80.0	79.9	80.3	80.7	80.9	81.3
Inpatient unit cost (per person per day) †		(JPY)	15,814	18,615	19,363	23,128	23,732	25,856	28,169	30,690
Outpatient unit cost (per visit) †		(JPY)	6,790	6,791	8,743	9,154	8,082	8,082	8,082	8,082
The number of public LTC insurance recipients ‡			838,510	856,321	864,439	857,859	749,622	685,107	623,608	566,692
Average nursing care level†			2.81	2.86	2.92	2.80	2.86	2.87	2.88	2.88

* Excerpted from "Population estimation."

† These were excerpted from government statistics (Future values were projected by linear approximation taking into account changes in population proportions based on statistics): "Estimated number of patients receiving medical treatment" and "Average length of stay" were taken from "the patient survey," "Number of deaths" and "Average age of death" were taken from "Vital Statistics," “Hospitalization unit cost” and “Outpatient unit cost” were taken from “Statistics of Medical Care Activities in Public Health Insurance,” and "Average nursing care level" was taken from "Comprehensive Survey of Living Conditions."

‡ Calculated from "Comprehensive Survey of Living Conditions" and "Statistics of Long-term Care Benefit Expenditures" (future values were projected by linear approximation taking into account changes in population proportions based on those two statistics).

**Table 2:** An overview of the results: The comprehensive cost of illness, past estimations and future projections.

			2008	2011	2014	2017	2020	2023	2026	2029
			**Cancer**							
Medical costs			9,115	9,847	9,993	10,537	10,248	10,099	9,931	9,804
	Medical direct cost		2,227	2,419	2,816	3,169	3,098	3,230	3,335	3,450
	Morbidity cost		481	475	463	461	415	385	357	334
	Mortality cost		6,406	6,953	6,713	6,907	6,735	6,484	6,239	6,020
LTC costs [OCA]			224	297	314	436	412	429	443	454
	Formal care cost		103	140	146	211	197	209	221	231
	Informal care cost [OCA]		121	157	168	225	214	219	222	223
Comprehensive Cost of Illness [OCA]			9,339	10,144	10,307	10,973	10,659	10,528	10,374	10,258
LTC costs [RA]			-	-	-	-	537	563	585	602
	Informal care cost [RA]		-	-	-	-	339	353	364	371
Comprehensive Cost of Illness [RA]			-	-	-	-	10,784	10,662	10,516	10,406
			**Heart diseases**							
Medical costs			3,797	4,125	4,114	4,596	4,537	4,576	4,578	4,649
	Medical direct cost		1,265	1,305	1,514	1,757	1,718	1,836	1,934	2,074
	Morbidity cost		187	195	185	199	162	147	132	120
	Mortality cost		2,346	2,624	2,415	2,640	2,656	2,593	2,512	2,456
LTC costs [OCA]			407	420	567	679	646	662	673	681
	Formal care cost		173	186	259	304	296	310	323	334
	Informal care cost [OCA]		234	233	308	375	350	352	350	347
Comprehensive Cost of Illness [OCA]			4,204	4,545	4,681	5,274	5,183	5,238	5,251	5,330
LTC costs [RA]			-	-	-	-	851	878	897	910
	Informal care cost [RA]		-	-	-	-	555	568	574	576
Comprehensive Cost of Illness [RA]			-	-	-	-	5,388	5,454	5,475	5,560
			**Cerebrovascular diseases**							
Medical costs			3,424	3,400	3,212	3,369	3,083	2,883	2,666	2,482
	Medical direct cost		1,436	1,367	1,444	1,511	1,420	1,390	1,339	1,299
	Morbidity cost		378	351	322	314	278	247	217	191
	Mortality cost		1,611	1,682	1,446	1,545	1,385	1,247	1,111	992
LTC costs [OCA]			3,152	3,300	3,484	3,170	2,794	2,486	2,205	1,955
	Formal care cost		1,495	1,608	1,733	1,551	1,383	1,246	1,120	1,005
	Informal care cost [OCA]		1,657	1,691	1,752	1,618	1,410	1,239	1,085	950
Comprehensive Cost of Illness [OCA]			6,576	6,700	6,696	6,539	5,876	5,369	4,871	4,437
LTC costs [RA]			-	-	-	-	3,618	3,246	2,899	2,584
	Informal care cost [RA]		-	-	-	-	2,235	2,000	1,779	1,579
Comprehensive Cost of Illness [RA]			-	-	-	-	6,701	6,129	5,565	5,066

(Billion JPY)

[OCA]: Cases where informal care costs are estimated using the opportunity cost approach (OCA), the standard method. OCA assumes that the family care system will continue in the future.

[RA]: Cases where informal care costs were estimated using the replacement approach (RA). RA assumes that all family caregivers are replaced with professional caregivers.

Regarding future projections and time trend, the total C-COI of cancer increased slightly until 2017, after which it was expected to show a slight decrease until at least 2029. It has been projected that the MDC of cancer would continue to increase, but the MbC and MtC declines would outweigh them in the future. It was estimated that the total C-COI of heart disease would continue to increase slightly. The breakdown was predicted to be an increase in MDC and slight decrease in MbC and MtC. Previously, the LTC costs for heart diseases have been on the rise but were estimated to level off in future projections. The total C-COI of CVD increased in 2014 compared with that in 2008. However, it began to decrease in 2017 and was predicted to continue to decrease thereafter. In future projections, all five CVD components are estimated to decrease. Regarding the details of the change in the LTC costs of CVD, both FCC and ICC showed an upward trend until 2014. However, it began to decrease in 2017.

The future projections for the ICC of CVD using RA were 1.58 to 1.66 times larger than those using OCA. Consequently, the ICC projected using RA accounted for 61.1%–61.8% of the LTC costs.

## Discussion

In this study, past estimations and future projections of the C-COI for the three major diseases in Japan were made using government statistics. Trends in the total amount and breakdown of C-COI showed different trends for each of the three diseases. The COI of cancer and heart diseases has been increasing to date, but the C-COI of cancer is predicted to start declining in the future. The C-COI of CVD has remained unchanged to date, but future projections suggest that it will decrease. The LTC costs accounted for a small proportion of the total C-COI of cancer and heart diseases but approximately half of the C-COI of CVD.

The C-COI for cancer was on the rise until 2017. The main contributor to this increase was the MDC. Table 1 shows that the number of cancer patients is estimated to continue to increase consistently from 2008 until 2029, and that the IUC and OUC will continue to rise. The rise in the MDC of cancer was considered to be due to these factors and was estimated to continue to rise in future projections. However, in future projections, it was estimated that the total C-COI of cancer will show a decreasing trend from 2020 onwards. This is because MtC, which accounts for the majority of the total C-COI of cancer, starts to decline. MtC accounted for a higher proportion of C-COI in cancer than in the other two diseases. This is because the age of death from cancer is the lowest of the three diseases and the human capital value lost is high. However, the average age of death from cancer is rising rapidly (Table 1, Fig 1). The decrease in MtC in cancer is attributed to a reduction in human capital loss due to an increase in the age of death. The MbC of cancer also declines as the average age of patients increases, reducing the lost labor value. The increase in the average age of patients and the prolongation of cancer patients’ lives due to advances in treatment methods may also have contributed slightly to the increase in MDC due to higher patient numbers and higher unit costs of treatment. However, they reduced MbC and MtC more than the increase in MDC, resulting in a reduction in the total C-COI of cancer.

The C-COI of heart disease was estimated to continue to increase consistently from 2008 to 2019. However, MtC, the largest component of the C-COI of heart diseases, is estimated to start declining in 2023, even though the number of deaths will continue to increase. The decline in MtC is thought to be due to an increase in the age of death, showing a trend similar to that of cancer. As the average age of death for heart disease is higher than that for cancer (Fig 1), the human capital value of deaths due to diseases is also lower. Therefore, the decline in MtC in heart diseases was expected to be more gradual than that in cancer, even though the percentage increase in deaths between 2020 and 2029 due to both diseases was not very different, +5.0% and +7.0%, respectively. MbC was also projected to decline in the future, as the number of patients would start to decline slightly and the proportion of older patients would increase (Table 1). At the same time, MDC was predicted to continue to increase owing to increases in the unit cost of treatment, particularly IUC.

CVD had a notably different proportion of components compared to the other two diseases. Estimates using OCA, the basic method, show that LTC costs account for approximately half of the C-COI, and MtC accounts for a smaller proportion of the C-COI than the other two diseases. This difference may reflect the long-term sequelae of CVD [5]. According to government statistics, CVD accounted for 16.6% of all "diseases that caused public LTC insurance certification” in 2016, whereas heart diseases and cancer, which have more patients, only accounted for 4.6% and 2.4%, respectively [31]. It is thought that people with CVD receive LTC insurance benefits more than those with the other two diseases. The breakdown of medical costs also differs between CVD and the other two diseases, with a higher proportion of costs allocated to MDC and a lower proportion to MtC. This may reflect the small number of deaths among those affected by CVD and the older average age of death. CVD has long been the leading cause of death in Japan, but its importance as a cause of death is now declining. Over time, the C-COI of CVD remained roughly flat until 2017; however, all components were projected to trend downward in the future. Previous studies on GBD reported that between 1990 and 2016, DALYs for CVD in Japan did not change dramatically, but age-adjusted DALYs decreased by > 50 percent [7, 32]. This suggests that, although the patient population has been aging, the apparent burden has not changed drastically until now. The C-COI estimates in this study up to 2017 were similar. However, future projections suggest a clear downward trend spurred by a further increase in the age of morbidity and mortality, as well as a decrease in the number of patients and deaths. Prevention of CVD, which causes long-term sequelae, has been a major health policy issue in Japan. Accordingly, the government and healthcare professionals have been working on reducing salt intake and smoking [33], which are major risks and causal factors of the disease. These policy efforts have been successful in reducing age-adjusted morbidity and mortality of CVD, which should lead to a reduction in the social burden of the disease in the future. It was suggested that future health policies should consider the allocation of healthcare resources in anticipation of a reduced social burden of CVD.

A breakdown of the LTC costs for CVD shows that informal care currently contributes to the same burden as formal care in monetary terms. In 2000, Japan introduced a public LTC insurance system in addition to conventional UHC to socialize the LTC that the family had been conducting until then. Nevertheless, it became clear that approximately half of the LTC costs incurred owing to CVD were sustained by family care. Family members providing informal care in Japan are also aging. According to the Comprehensive Survey of Living Conditions, the average age of family caregivers in 2016 was 65.5 years, and the proportion of those aged 60 years and over reached 70% [31]. Table 2 shows that the share of ICC in the LTC costs gradually decreased. This may be due to the aging of family caregivers, which has reduced the loss of labor value. In other words, ICC can be viewed as being compressed by the care provided by older family caregivers. However, the further aging of family caregivers may make it difficult to maintain the current family care system and increase the demand for professional caregivers. As of 2017, the average wage of professional caregivers in Japan was 1,666 JPY, which exceeds the average labor value of older adults, who constitute the core generation of family caregivers [34]. Regarding future projections for CVD, the ICC by the RA, which assumed that all family caregivers would be replaced, was 1.58 to 1.66 times higher than that by the OCA, which assumed that the family care system would be maintained. In the future, the ICC may rise and advance from OCA to RA estimates as the transition from family caregivers to professional caregivers progresses. Although the social burden of CVD is expected to decrease in the future, there is still a need to prepare for rising ICC, and policy considerations to reduce or replace the burden on older family caregivers are needed. Perhaps, a shift in resource allocation from medical care to LTC should be considered.

These methods for future projections have some limitations. In this study, future projection of each variable was performed using linear or logarithmic/exponential approximations. No social factors were expected to suppress the increase in unit costs for medical care. Therefore, future projections were made under the assumption that the IUC and OUC would consistently increase. In particular, the IUC, which has a trade-off relationship with ALOS, was assumed to show an exponential increase. In recent years, although ALOS has been rapidly decreasing owing to the policy of the Japanese government [35], the procedure and medication on admission have not decreased to the same extent; consequently, IUC seems to have increased. While these assumptions are temporarily valid, they are not suitable for long-term forecasts because they can be violated by major changes in social conditions and the environment.

In addition, it was difficult to correct errors and misclassifications in the government statistical data. For example, it cannot be ruled out that deaths due to comorbidities were misclassified as deaths from the three major diseases. In this study, data from vital statistics were used to identify the causes of death. The vital statistics are compiled on the basis of death certificates issued by physicians who know the course of the patient leading to death, except in some exceptional cases. Japanese death certificates are formatted to provide a sequential and detailed description of the causal factors leading to death, which is expected to produce relatively accurate cause-of-death statistics [36]. All statistics on the cause of death by the Japanese government are based on death certificates and are the most reliable data on cause of death available in Japan. Even if some misclassifications occur during the statistics, they are unlikely to seriously affect the results of this study, which are macro-estimates.

In this study, as data from public LTC insurance are used to estimate LTC costs, it is not possible to consider patients who have sequelae from disease but do not use the LTC insurance system. Public LTC insurance is supposed to be secured by all older people aged 65 and over, but to actually receive the subsidy, an application must be made. By applying to the local government and receiving certification, 90% of the fees for LTC services can be subsidized by the insurance system. Those who need LTC but do not use public LTC insurance are likely to be limited to people with exceptional circumstances, such as ignorance of the system or a lack of capacity to apply. Therefore, this limitation was not expected to majorly impact the overall projection results. Although it is difficult to ascertain the number of such people since the establishment of public LTC insurance, the proportion of the older adult population using LTC insurance has been on the rise, particularly among people with mild disabilities [4]. This may indicate that public LTC insurance schemes have become more widespread. Therefore, this limitation is unlikely to have had a major impact on the overall results.

In addition to the C-COI method, numerous attempts have been made to explore the future value of the social burden of the disease. For example, previous studies have shown that public health expenditure is procyclically influenced by the macroeconomic environment and exhibits cyclical behavior [37–40]. Using this approach, it may be possible to forecast public health expenditure over the business cycle. However, these methods are not suitable for projecting the burden of the disease in Japan. UHC is available to all citizens in Japan; it covers almost all treatments, including expensive treatments and medications, and is available in all hospitals, including private ones [41]. Newly developed treatments are quickly covered by UHC. Out-of-pocket expenses are set at no more than 30% of the total amount and are further reduced, especially for people with low incomes and older adults. Furthermore, there is a cap on out-of-pocket expenses based on income, such that even if a patient receives expensive treatment, all costs above a certain amount are covered by UHC. Due to this background, Japanese citizens are rarely deterred from visiting hospitals for economic reasons, and public healthcare expenditure in Japan is considered to be less affected by the business cycle than in the countries dealt with in previous studies. A World Bank work paper also states that health expenditures in Japan have escaped the effects of the business cycle [42].

Attempts to estimate the burden of LTC, including informal care, have also been made worldwide [43–50]. These studies suggest that the burden of LTC, especially informal care, cannot be ignored, even in countries outside Japan. However, the definition of indirect costs varies, and a standardized method is yet to be developed. In addition, many previous studies have used a small sample for cost calculations. The C-COI method and the method of future projections provide a clear and comprehensive estimate of the burden borne by the country as a whole. Despite some limitations, these methods seem to be suitable for estimating the social burden of disease macroscopically, while taking advantage of Japan’s extensive government statistics, including those related to LTC. Rapid aging is predicted in many Asian and European countries in the near future [51]. As COI methods have already been studied in many countries around the world, it seems feasible to apply C-COI-based methods for future projections of the social burden of disease, including LTC, to other countries where data are available.

## Conclusion

The C-COI method was used to visualize the burden of medical care and LTC, including indirect costs, from the past to the future. The C-COI of the three major diseases differed greatly in their total amount, components, and trends over time. For cancer and heart diseases, MDC is projected to continue to increase, whereas MbC and MtC are projected to decrease due to increases in the age of onset and death. This suggests that the total social burden of cancer, which is the leading cause of death in Japan, will decline in the future. The proportion of LTC costs in the C-COI of CVD, which has long been a major health issue in Japan as a chronic disease causing long-term sequelae, remains very high compared to that of the other two diseases; however, the overall social burden is expected to decrease in the future. At the same time, further aging of family caregivers could lead to the collapse of the current family care system and increase costs through a substantial increase in the demand for professional caregivers. Policies may be needed to shift resource allocation from medical care to LTC.
